# An unusual pathological finding of chronic lymphocitic leukemia and adenocarcinoma of the prostate after transurethral resection for complete urinary retention: case report

**DOI:** 10.1186/1471-2407-4-95

**Published:** 2004-12-22

**Authors:** Riccardo Ballario, Paolo Beltrami, Stefano Cavalleri, Lorenzo Ruggera, Maria Grazia Zorzi, Walter Artibani

**Affiliations:** 1 Divisione Clinicizzata d'Urologia, University of Verona, Verona, Italy; 2 Anatomia Patologica, University of Verona, Verona, Italy

## Abstract

**Background:**

We describe a patient who underwent transurethral resection of the prostate for urinary obstructive symptoms and had histological findings of adenocarcinoma of the prostate with prostatic localization of chronic lymphocitic leukemia (CLL).The contemporary presence of CLL, adenocarcinoma of the prostate and residual prostatic gland after transurethral resection has never been reported before and the authors illustrate how they managed this unusual patient.

**Case presentation:**

A 79-years-old white man, presented with acute urinary retention, had a peripheral blood count with an elevated lymphocytosis (21.250/mL) with a differential of 65.3% lymphocytes and the prostate-specific antigen (PSA) value was 3.38 ng/mL with a percent free PSA of 8.28%. The transrectal ultrasound (TRUS) indicated an isoechonic and homogenic enlarged prostate of 42 cm^3 ^and the abdomen ultrasound found a modest splenomegaly and no peripheral lymphadenophaty. The patient underwent transurethral resection of the prostate and had a pathological finding of adenocarcinoma in the prostate with a Gleason Score 4 (2+2) of less than 5% of the material (clinical stage T1a), associated with a diffused infiltration of chronic lymphocitic leukemia elements.

**Conclusions:**

The incidental finding of a prostatic localization of a low-grade non-Hodgkin's lymphoma does not modify eventually further treatments for neither prostate cancer nor lymphoma. The presence of a low-grade and low-stage lymphoma, confirmed by a hematological evaluation, and the simultaneous evidence of an adenocarcinoma after transurethral resection of the prostate for acute urinary retention do not require any immediate treatment due to its long-term survival rate and the follow-up remains based on periodical PSA evaluation and complete blood count.

## Background

The incidence of non-Hodgkin's lymphoma among prostate cancer patients is extremely low (0.2%), and, even more the leukemic infiltration of the prostate [1,2]. It has been reported that a chronic lymphocitic leukemia (CLL) may have its first clinical manifestation with acute urinary retention [3]. We describe a patient who underwent transurethral resection of the prostate for urinary obstructive symptoms and had histological findings of adenocarcinoma of the prostate with prostatic localization of CLL. The contemporary presence of CLL, adenocarcinoma of the prostate and residual prostatic gland after transurethral resection has never been reported before and the authors illustrate how they managed this unusual patient.

## Case presentation

**Figure 1:**
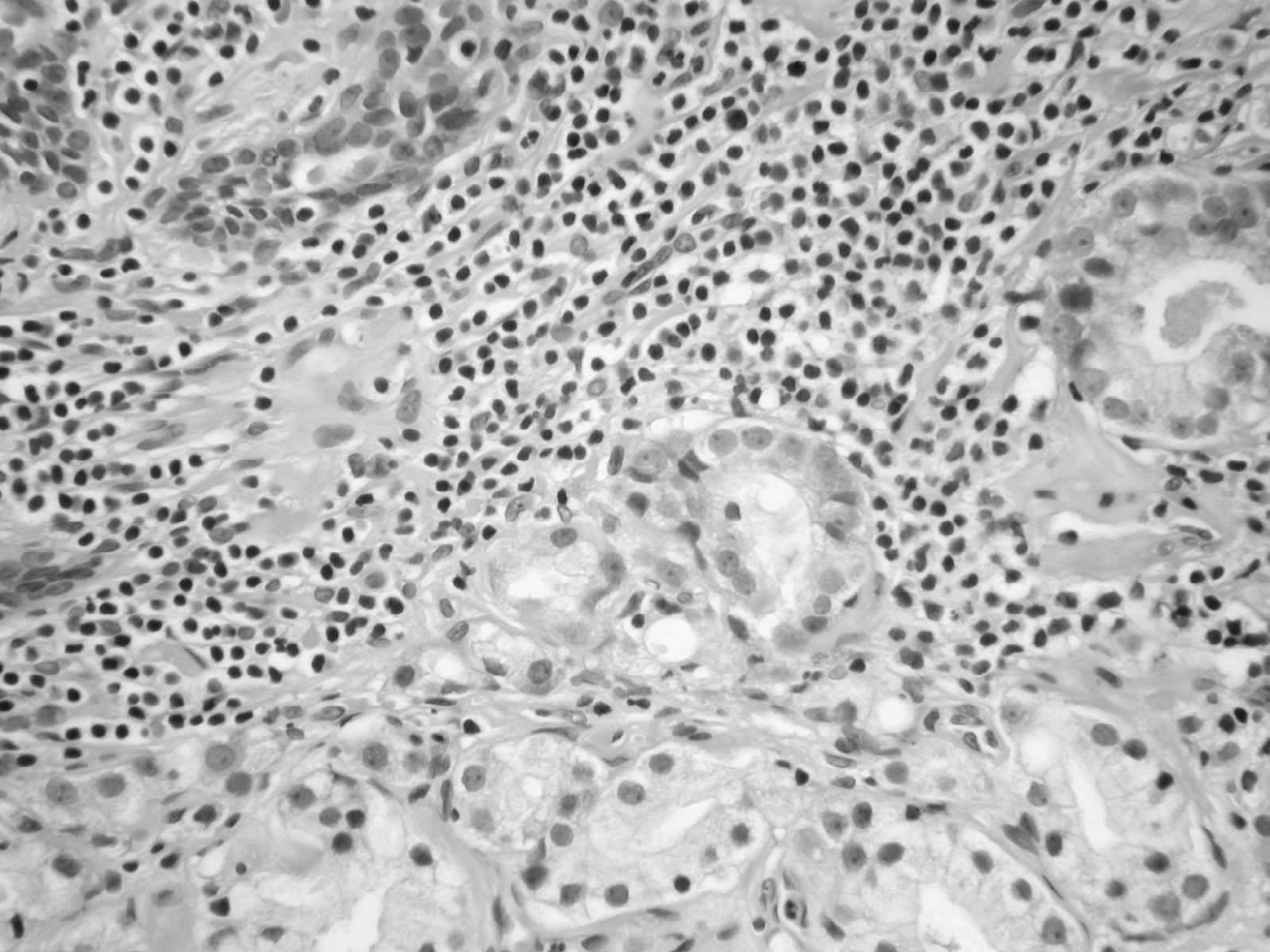
Area of surgically removed prostate demonstrating microfoci of adenocarcinoma associated with a localization of small-B cell lymphoma (CLL) (EE, 40X).

**Figure 2:**
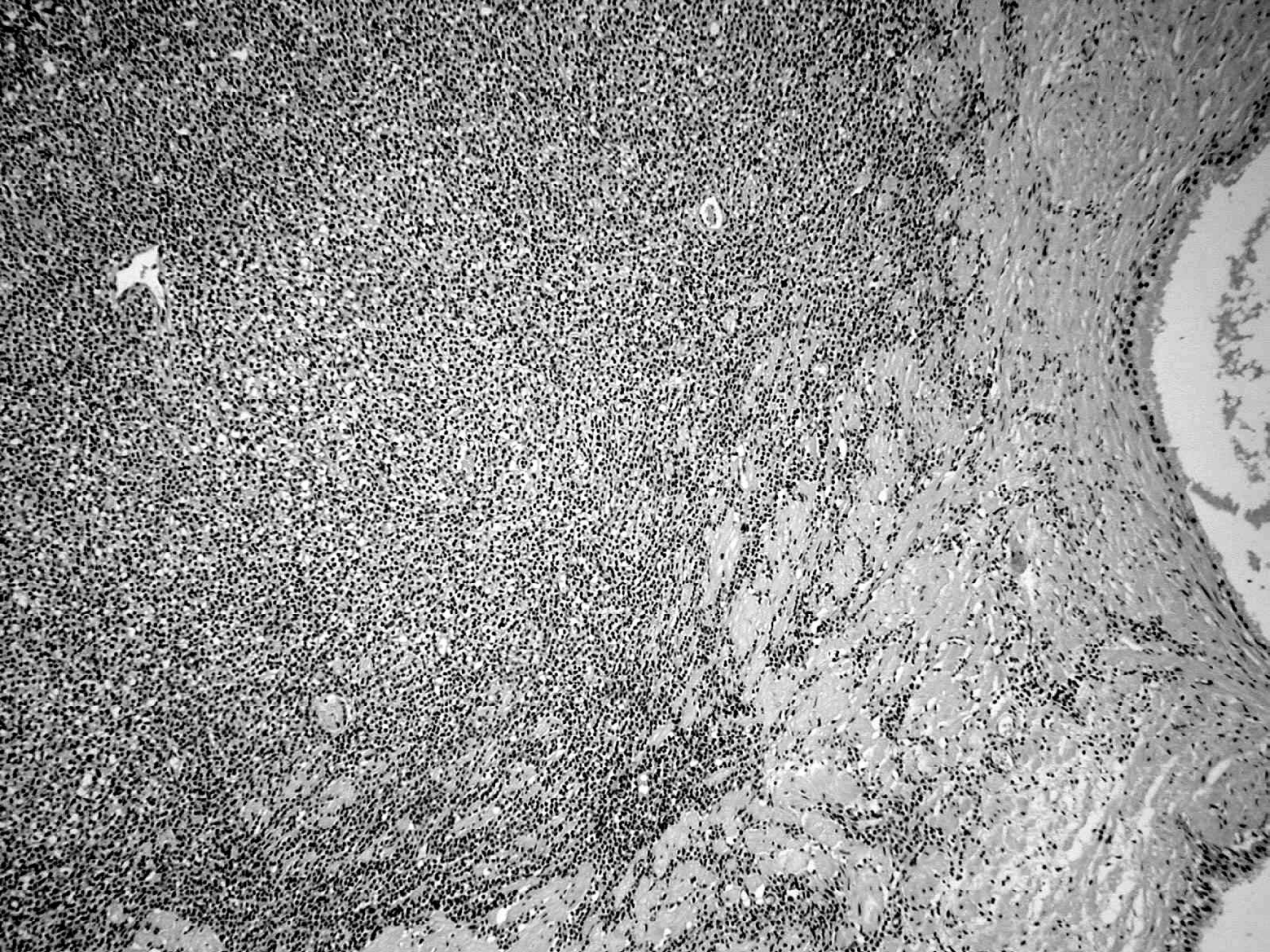
Area of surgically removed prostate with localization of chronic lymphocitic leukemia (CLL) (EE, 10X).

A 79-year-old white man, presented with acute urinary retention, was initially treated by indwelling catheter and was referred for complete urological evaluation. The digital rectal examination revealed a benign feeling prostate of 40 g, the physical examination was negative and the International Prostatic Symptoms Score (I-PSS) was 21. The transrectal ultrasound (TRUS) indicated an isoechonic and homogenic enlarged prostate of 42 cm^3 ^and the abdomen ultrasound found a modest splenomegaly and no peripheral lymphadenophaty. The peripheral blood count showed an elevated lymphocytosis (21.250/mL) with a differential of 65.3% lymphocytes, while other parameters as platelet count, number of erythrocytes and lactate dehydrogenase hormone level were all normal. The prostate-specific antigen (PSA) value was 3.38 ng/mL with a percent free PSA of 8.28%. Due to his age and symptoms, the patient underwent transurethral resection of the prostate. The pathological examination of the tissue revealed an adenocarcinoma in the prostate with a Gleason Score 4 (2+2) of less than 5% of the material (clinical stage T1a), associated with a diffused infiltration of chronic lymphocitic leukemia elements (see Figure 1), CD5 and CD20 positive (see Figure 2). The patient underwent a computed tomography of the abdomen, confirming only a moderate splenic enlargement but not lymphadenomegaly (stage 2 of Rai) and was then referred for a haematological evaluation. No other investigations or treatments were required due to the age, the low grade and stage of the disease in this patient. At a follow-up of 24 months the patient is alive, with a PSA value of 0.65 ng/ml, a stable haematological condition and the International Prostatic Symptoms Score (I-PSS) is 6.

## Conclusions

The prostatic involvement by non-Hodgkin's lymphomas is an unusual finding. The simultaneous presence in the prostate of CLL and adenocarcinoma has been reported in a percentage variable from 0% (4319 patients underwent radical prostatectomy in Eisemberger *et al.*,) to 0.8% (1092 patients underwent radical prostatectomy in Terris *et al.*) [1,2]. It has been demonstrated that the presence of malignant lymphocitic infiltrate in the prostate cannot be detected on TRUS, and the ultrasound-guided prostate needle biopsies exhibit a lack of diagnostic accuracy with a sensitivity of only 22% for detecting leukemic infiltration of the prostate [4]. In our case, the ultrasound images showed only a modest splenomegaly, with no other specific finding, and the low value of PSA with the age of the patient did not indicate for prostate biopsies and eventually consequent radical prostatectomy. The patient underwent transurethral resection on the basis of his voiding symptoms. The presence of a low-grade and low-stage lymphoma, confirmed by a haematological evaluation, and the simultaneous evidence of an adenocarcinoma after transurethral resection of the prostate for acute urinary retention, do not require any immediate treatment due to their long-term survival rate and the follow-up should be based on periodical PSA evaluation and complete blood count [5]. The incidental finding of a prostatic localization of a low-grade non-Hodgkin's lymphoma, even in presence of a residual prostatic gland, does not modify eventually further treatments for neither prostate cancer nor lymphoma.

## Competing interests

The author(s) declare that they have no competing interests.

## Author's contributions

RB and PB managed the patient, edited the manuscript and coordinated the submission. LR carried out the literature search, SC performed the surgical procedure and MZ performed the pathological examination and realised the figures. WA revised the manuscript for scientific content. All authors contributed to the preparation of the manuscript. All the authors read and approved the final manuscript.

## Pre-publication history

The pre-publication history for this paper can be accessed here:
